# Perspectives, attitudes and experiences of introducing noninvasive medical technologies in end-of-life care: a scoping review

**DOI:** 10.1186/s12904-025-01890-4

**Published:** 2025-09-29

**Authors:** Laila Hov, Reidun Førde, Simen Steindal, Hilde Tinderholt Myrhaug, Ingrid J. Skogestad, Bjørn Erik Juel

**Affiliations:** 1 VID Specialized University, Diakonveien 12-14, Oslo, 0370 Norway; 2 University of Oslo, Gaustadalléen 21, Oslo, 0349 Norway; 3 OsloMet, Pilestredet Park, Oslo, 0890, 0176 Norway; 4 Vestre Viken HF, Drammensveien 4, 3612 Kongsberg, Norway

**Keywords:** End-of-life care, Palliative care, Palliative medicine, Noninvasive technology, Review

## Abstract

**Background:**

There is a strong tradition for withdrawing medical equipment from patients receiving end-of-life (EOL) care. However, the introduction of new noninvasive medical equipment could be acceptable provided that it improves the quality of care and patient comfort.

**Objectives:**

We aimed to systematically map published studies of the perspectives, attitudes and experiences of health care professionals, dying patients, and their next of kin, about introducing noninvasive medical technology in end-of-life care.

**Design:**

We conducted a scoping review. On January 27, 2025, systematic searches of five databases were carried out (Medline (EBSCO), CINAHL, Embase, Academic Search Elite, and Cochrane Library CDSR). Inclusion criteria were empirical studies published in English or Scandinavian languages. We searched for qualitative, quantitative or mixed method studies focusing on perspectives on, experiences of and attitudes toward introducing noninvasive medical technologies in end-of-life care from the view of health care professionals, dying patients, and their next of kin. Three pairs of researchers independently assessed potential eligibility and data extraction. Data would be summarized qualitatively.

**Results:**

The searches yielded 3288 unique articles, 3194 of which were excluded following an initial screening. Among the remaining 94 articles, none satisfied our inclusion criteria. Thus, we found no empirical articles addressing perspectives on, attitudes toward, or experiences with the use of noninvasive medical technology in end-of-life care.

**Conclusions:**

Empirical research exploring the perspectives on, attitudes toward, and experiences of, and use of noninvasive medical technology in end-of-life care is needed. This is important for understanding when and how it is feasible, useful, and ethical to introduce such technologies in the terminal phase of palliative care.

**Supplementary Information:**

The online version contains supplementary material available at 10.1186/s12904-025-01890-4.

## Introduction

End-of-life (EOL) care aims to provide a “good death” by alleviating distressing symptoms such as pain, dyspnea, and anxiety [1–3]. However, in the final days of life, many patients become entirely unresponsive [4], making symptom assessment challenging. This is particularly true for patients receiving palliative sedation, where pharmacological interventions intended to relieve suffering further reduce their ability to communicate distress [5, 6].

Although behavioral unresponsiveness is typically associated with unconsciousness, it is well established that subjective experience can persist even in unresponsive states [7–10]. If this goes unnoticed, it can lead to unnecessary distress and suffering, highlighting the need for improved methods to assess consciousness in those who can no longer respond behaviorally [11–18]. In EOL care settings, where standard assessments rely heavily on observable behavior because the use of technology challenges the normative understanding of ‘dying with dignity’ [19], it is possible that some unresponsive patients retain consciousness covertly. Among dying patients who could still communicate, over half experienced dyspnea, nearly half suffered from pain, and nearly one-third reported anxiety [20]. Encapsulated, it is conceivable that a large proportion of unresponsive patients in EOL care continue to suffer despite having lost the capacity to tell anyone about it. At the same time, it is of great interest for the family to be informed of the patient’s mental presence.

One potential solution lies in objective brain monitoring, particularly electroencephalography (EEG), owing to its ease of use and noninvasive nature. EEGs are widely used in clinical and research settings to monitor patients in situations where the correlation between responsiveness and consciousness is disrupted, such as disorders of consciousness [21, 22], delirium [23–25], anesthesia [26–28], sleep [29–31], and epilepsy [32]. The technique is noninvasive, cost effective, and feasible for bedside use, making it suitable for EOL care settings [33]. Preliminary research suggests that EEG-based assessments could provide valuable insights into the conscious state of dying patients [34], potentially improving symptom management [35]. However, while EEG has been successfully implemented in other medical contexts, its application in EOL care remains limited.

Despite the potential benefits of using noninvasive medical technology in end-of-life care, the study of dying patients remains sensitive and is often considered controversial [36]. Recruiting such patients to research at the final stages of life is known to be a persistent challenge in EOL care research [37]. The emotional weight of EOL care for next of kin, ethical concerns, and cultural attitudes contributes to a longstanding reluctance to systematically investigate the experiences of unresponsive patients and their caregivers. This hesitancy has likely contributed to a significant knowledge gap regarding the acceptability and feasibility of introducing such technologies in EOL care settings. A heedful approach is necessary under these circumstances. For instance, in an ongoing research project, validated EEG measures of consciousness will be used for bedside monitoring of the conscious state of dying patients [38]. To justify such an intervention ethically, it is important to understand how dying patients, their next of kin, and healthcare professionals’ the use of noninvasive technologies at the EOL. Performing a broad literature review (such as a scoping review), including published studies with various research methods and the perspectives, attitudes and experiences of health care professionals, dying patients, and their next of kin, is important to achieve a more comprehensive understanding. Such knowledge could be important to understand whether empirical studies applying noninvasive medical technology in the EOL setting is ethically permissible. Furthermore, a scoping review could identify research gaps and determine whether it is feasible to conduct a systematic review [39]. Consequently, this scoping review aimed to systematically map published studies of the perspectives, attitudes and experiences of health care professionals, dying patients, and their next of kin, about introducing noninvasive medical technology in EOL. The following research question guided the review: What is known from exiting empirical studies about the attitudes, perspectives, and experiences of healthcare professionals’, terminal patients, and their next-of-kin regarding the use of non-invasive medical technologies in EOL setting?

## Methods

This scoping review employed the methodological framework by Arksey and O’Malley [40], which consists of the following stages: identifying the research question; identifying relevant studies; study selection; charting the data; and collating, summarizing, and reporting the results. We also employed updated methodological guidance for conducting scoping reviews [39, 41]. The review was reported in accordance with the Preferred Reporting Items for Systematic Reviews and Meta-Analyses extension for Scoping Reviews (PRISMA-ScR) checklist [42]. The review protocol was registered on the Open Science Framework (https://osf.io/9pyfw/).

### Eligibility criteria

The eligibility criteria are described in Table 1 using the population, concept and context framework as well as the type of studies, language and period.

**Table 1:** Eligibility criteria

Criterion	Inclusion	Exclusion
Population	Patients, next of kin, or healthcare professionals	
Concept	Perspectives, attitudes, or experiences of the introduction of noninvasive medical technology in imminently dying patients	
Context	Any type of healthcare institution	
Type of studies	Quantitative, qualitative or mixed-method studies published in scientific peer-reviewed journals	Any type of reviews, protocols, commentaries, editorials, letters, opinion pieces, case-studies, guidelines, master’s thesis, PhD thesis, books or book chapters
Language	English or Scandinavian	All other languages
Period	From inception to January 27th 2025	After January 27th 2025

We had no limitations regarding period, as we wanted to map published empirical studies [41], and research articles were limited to full-text articles written in English or Scandinavian languages, as all the reviewers understood these languages.

### Search

We worked closely with experienced librarians (Hannah Pope and Anna Kirsten Nygaard) to develop the search strategy. We initially employed a broader search strategy. Following a pilot screening of 1,000 abstracts, in December 2023 to test the search strategy and eligibility criteria, and subsequent meetings among the authors and with librarians, we decided to exclude articles involving COVID-19. A pilot search was conducted, resulted in refinement of both the search terms and the eligibility criteria. The final search strings for all the databases can be found in Appendix 1.

### Information sources

The librarians searched Medline (EBSCO), CINAHL, Embase, Academic Search Elite, and Cochrane Library CDSR on February 12, 2024, and conducted an updated search on January 27, 2025.

### Selection of the sources of evidence

The search results were transferred to EndNote for the removal of duplicates. The search results were subsequently transferred to the webtool Rayyan [43] for study selection. Three pairs of reviewers independently screened titles and abstracts for relevance. Any disagreement was resolved within the pair. After screening the first hundred search results independently, all six reviewers met to discuss their evaluations to reduce variability between reviewers in the subsequent screening. Three reviewers independently assessed the resulting articles in full text against our eligibility criteria, and any disagreements were resolved through discussion.

### Data extraction and charting

We planned to develop a standardized charting form in Word/Excel. The form would include the essential information such as author, year, and country. In addition: aim; sample and setting, design, technological solution. use of technology and findings relevant for the research question. The data charting form would be piloted on some studies to assess whether it needed to be revised. One author would extract data, while another author would check data accuracy against the articles (https://osf.io/9pyfw/).

### Synthesis

We planned to summarize the data from the results and discussion sections of the included articles using a qualitative approach following the steps developed by Arksey and O’Malley [39].

## Results

**Fig. 1:**
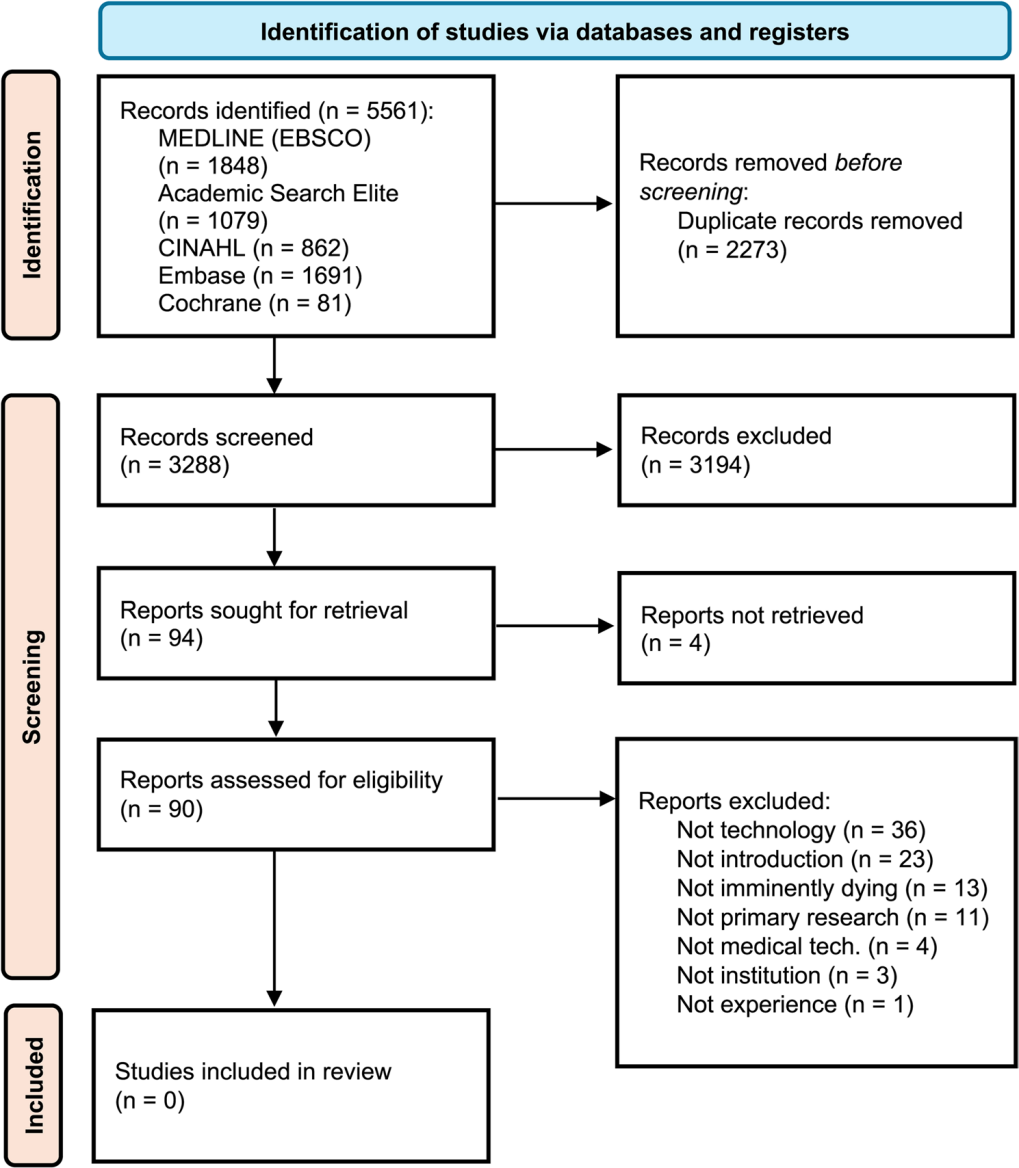
Flowchart of the screening process

The database searches yielded 5561 articles across the five databases. After removing duplicates (*n* = 2273), the titles and abstracts of 3288 records were screened. The full texts of 94 articles were assessed. None of the full-text articles met our inclusion criteria. The main reasons for the exclusion of full-text articles were (1) perspectives, experience and attitudes were not included in the dataset (“not focused on experience”), (2) technology was not within the scope (“not focused on technology”), (3) the study was conducted in home-based care (“not healthcare institution”), (4) imminently dying was not explicitly addressed (“not imminently dying”), (5) focused on the withdrawal of life-prolonging technology aimed at curing or stabilizing the patient (“not introduction of technology”), (6) telecommunication or sensory devices, not affixed to the patient (“not medical technology”), and (7) not relevant format (“not primary research”) (Fig. 1).

## Discussion

Our scoping review aimed to systematically map empirical studies of the perspectives, attitudes, and experiences of healthcare professionals, dying patients, and their next of kin, about introducing noninvasive medical technology in EOL care.

Our systematic search revealed that our empirical knowledge on the topic is limited. While the absence of eligible studies in this review might appear as a limitation, the fact that our review identified no empirical evidence from published studies on this topic is an important finding, highlighting a potentially important research gap. Such knowledge is important to understand whether the common EOL care practice of reducing technological load as patients approach death aligns with the perspectives and attitudes of those most affected by them.

We believe that the absence of published studies imply that the technological progress in improving patient care in other areas of nursing and medicine has not reached the field of EOL care, as problematized by White et al. [36]. They noted that research in this field is both challenging and controversial and that little is known about the viewpoints of dying patients and their next of kin. Additionally, as Kars et al. [37] noted, health care professionals work as gatekeepers in recruiting patients at the EOL to participate in research, which may also make the process of accessing this field more difficult. From our point of view, this becomes an ethical issue as well. We know that dying is a dreaded part of human life, and as such, it is pertinent to ensure that we provide state-of-the-art EOL care when patients are approaching death. Thus, there seems to be a need to ensure that technological development aiming to improve our knowledge of what takes place in the last hours of life is an area of importance that needs to be addressed.

In our search for articles, one study indirectly implied that dying while being attached to technology is forwarded as less peaceful and dignified than being without, but that sometimes next of kin found comfort in how technology such as heart monitoring provided information about the patient status [44]. For example, nurses may believe that EOL care is high-touch, rather than high-tech [45], and may also stand in the way for health care professionals to recognize patients as dying [46].

Furthermore, literature from related areas offers useful lessons. For example, there is evidence that digital tools such as telecommunication platforms or electronic records can help coordinate care and support patient centered practice, if they enhance rather than replace direct human contact [47]. In other words, technology is more acceptable when it is clearly an aid to everyday care and is a supplement rather than a substitute for it. It is conceivable that similar principles could guide the introduction of noninvasive medical technology in a way that can support the relational aspects of care in the EOL setting.

In one study, nurses reported inexperience with assessing symptoms near the EOL. Being able to identify subtle signs of agitation, pain and discomfort, are essential prerequisites for optimal EOL care and a necessary skill for nurses and physicians [48]. However, another study found that the electronic patient records of hospitalized dying patients often lacked assessment of pain intensity and according to the electronic patient records, 10% of the patients receive inadequate pain management [49]. It is conceivable that transient use of other sensor technologies (e.g. sweat, respiratory rate, and movements) for the purpose of provide information that is otherwise inaccessible to optimize symptom relief. For example, we know that the use of EEG technology in other fields has provided useful knowledge about patients’ states; noninvasive brain monitoring during general anesthesia is commonly used to ensure the patients are in a state suitable for surgery [50]. Such monitoring equipment may inform health care professionals not only about patients’ capacity to feel pain but also about the extent to which they can hear and understand their loved ones, despite their ability to communicate by normal means. Therefore, such technologies may provide information about the patient state that would otherwise remain unattainable. However, without high-quality, empirical research on the population in question, we may never know whether and to what extent noncommunicating patients in EOL covertly retain these capacities.

The strong tradition of “Total Care” in EOL settings reminds us that technology can unintentionally shift attention toward what is easily measured and away from the psychosocial and spiritual dimensions of care [51]. Analyses of smart sensor technologies highlight the need for careful, purposeful adoption that respects patient autonomy and maintains holistic care [51]. Furthermore, a study suggested that technologies mediate care in complex ways: they can make relational work less visible, but when used thoughtfully, they can also enhance attentiveness and support caregivers in ways that align with EOL values [52]. Such adjacent work may help provide a practical framework for how non-invasive medical equipment may be introduced responsibly and in harmony with the principles of EOL care.

We believe that distinguishing between technology as a contributor to overtreatment and noninvasive technology with the potential to improve symptom management is pertinent [35]. From our point of view, technology in itself is considered a source that disrupts peaceful dying—at least, technology other than what is used to administer sedatives and analgesics. We are well aware of the complexity of the situation and the need for a highly sensitive approach. However, we argue that these established practices influence our approach to conducting research involving dying patients [36, 37]. This may stand in the way of allowing technology to improve our knowledge of the dying process and thus our ability to care for our patients.

So, what kind of knowledge can we establish through an empty review? Obviously not the kind of knowledge that we hoped to find. Hence, we are not in a position to offer any strong conclusions regarding perspectives on, attitudes toward, and experiences with the introduction of noninvasive technology when the patient is dying. However, we argue that this empty review provides us with insight into several aspects that may shed light on why we could not find relevant studies. Yaffe et al. [53] conducted an analysis of Cochrane reviews with no included studies and described the following factors of what characterizes empty reviews: the novelty of the field, the circumstances of the study, such as population and context, or too rigid inclusion criteria. We believe that all these factors may be relevant in explaining our empty scoping review. The research team frequently discussed the possibility of widening the inclusion criteria to see if that would lead to relevant articles. However, we landed on keeping the criteria as they are.

There are several aspects of our methodology that should be considered for transparency. Our research question and inclusion criteria, focused on empirical studies of how users and stakeholders view the introduction of noninvasive medical technologies in the EOL setting, explains why no eligible studies were identified. A broader scope, for example including grey literature, studies of related technologies such as telehealth or electronic patient records, or work outside of the terminal stage, may have yielded more material for synthesis. However, we considered such broadening carefully and concluded that it risked diluting our central question.

There may be synonyms of both EOL and noninvasive medical technology that we may not have been able to identify and include in our search strategy. Our review also had some language limitations. Consequently, there may be studies we may not have been able to identify. Another limitation could be that we did not include grey literature, as we wanted to map published studies. However, it is the aim of the review that decides was type of studies or literature that should be included, and given the sensitivity of the field, we wanted to make sure to include studies that had been approved by registered ethical committees and peer reviewed. Furthermore, work in our team to best capture our research question, led to a search strategy that was broad with regards to the population [42] (patients, health care professionals, and next-of-kin) and experiences considered (perspectives, attitudes, and experiences) but narrow in terms of timeframe (imminently dying) and technologies (noninvasive medical technology attached to the patient).

Lastly, the term “non-invasive technology” in palliative care is frequently applied to a wide range of devices, including telemedicine solutions, mobile applications, artificial intelligence-driven support tools, and wearable sensors [47]. Our specific interest was to map research on wearable medical technologies; consequently, our use of “non-invasive technologies” reflects a narrower scope than what is typically described in the broader literature in this field.

## Conclusions

This empty scoping review indicates that there is no primary research revealing the perspectives, attitudes and experiences of health care professionals, patients, or their next of kin on the introduction of noninvasive technology in EOL care. While this makes sense given the views implicitly undergirding the prevailing tradition in EOL care, it is unfortunate if EOL care is to follow in the footsteps of modern medicine more broadly in adopting an empirically grounded practice. We believe that the lack of research on perspectives, attitudes and experiences regarding the use of noninvasive technology in EOL care relates to technology as such being seen as disruptive. We argue that this understanding may hamper the goal of providing personalized care for every patient as a unique individual. To remedy this, a conversation regarding the place of medical monitoring equipment in EOL care is warranted, and more empirical research is needed. Furthermore, it is not feasible to conduct a systematic review on this topic.

Future empirical studies should explore the perspectives, attitudes, and experiences of dying patients, next of kin and health care professionals regarding the introduction of noninvasive medical technology in EOL care.

## Supplementary Information

Supplementary Material 1.

## Data Availability

No datasets were generated or analysed during the current study.
