# Supplementary material for: Perspectives, attitudes and experiences of introducing noninvasive medical technologies in end-of-life care: a scoping review
Source: BMC Palliat Care. 2025 Sep 29;24:238. doi: 10.1186/s12904-025-01890-4 (PMC12482546; doi:10.1186/s12904-025-01890-4)
Supplement: Supplementary file 1 — Supplementary Material 1. [file 12904_2025_1890_MOESM1_ESM.docx]

Appendix 1: Search string

| Database: | Medline (EBSCO) |
| --- | --- |
| Filters: | Language (English, Scandinavian) Peer-reviewed articles |
| Search date: | 09.02.24/27.01.2025 |
| Number of results: | 1716 |

| **#** | **Query** | **Results** |
| --- | --- | --- |
| S1 | (MH "Respiration, Artificial+") | 90,044 |
| S2 | artificial n2 respirat* | 58,638 |
| S3 | respirat* | 1,063,531 |
| S4 | noninvasive ventilat* | 4,551 |
| S5 | ventilat* | 231,389 |
| S6 | vaporizer* | 11,300 |
| S7 | blood pressure device* | 2,276 |
| S8 | (MH "Electrical Equipment and Supplies+") | 221,160 |
| S9 | electroencephalograh* OR EEG | 126,169 |
| S10 | medical N2 (equipment OR intervention* OR technolog* OR device*) | 147,521 |
| S11 | (MH "Electrodes+") | 150,440 |
| S12 | electrode* | 237,217 |
| S13 | (MH "Oxygenators+") | 3,035 |
| S14 | oxygenator* | 5,461 |
| S15 | pulse oximeter* | 9,111 |
| S16 | oximeter* | 10,014 |
| S17 | S1 OR S2 OR S3 OR S4 OR S5 OR S6 OR S7 OR S8 OR S9 OR S10 OR S11 OR S12 OR S13 OR S14 OR S15 OR S16 | 1,787,554 |
| S18 | (MH "Terminal Care+") | 57,734 |
| S19 | terminal care | 39,239 |
| S20 | end of life | 46,646 |
| S21 | dying | 41,657 |
| S22 | dying process | 947 |
| S23 | (MH "Hospice Care") | 8,213 |
| S24 | hospice care | 34,018 |
| S25 | (MH "Palliative Care") | 64,173 |
| S26 | palliative care | 100,383 |
| S27 | terminal* n2 patient* | 9,713 |
| S28 | S18 OR S19 OR S20 OR S21 OR S22 OR S23 OR S24 OR S25 OR S26 OR S27 | 193,813 |
| S29 | (MH "Caregivers") | 51,893 |
| S30 | carer* | 48,575 |
| S31 | caregiver* | 112,188 |
| S32 | (MH "Family+") | 374,160 |
| S33 | famil* | 1,758,330 |
| S34 | loved one* | 5,291 |
| S35 | partner* | 292,606 |
| S36 | spouse* | 36,006 |
| S37 | significant other | 11,773 |
| S38 | next of kin | 1,856 |
| S39 | relative* | 1,706,556 |
| S40 | (MH "Medical Staff+") | 29,049 |
| S41 | medical staff | 43,231 |
| S42 | healthcare provider* | 71,817 |
| S43 | healthcare professional* | 43,295 |
| S44 | healthcare worker* | 56,129 |
| S45 | (MH "Medical Staff, Hospital+") | 26,037 |
| S46 | (MH "Nurses+") | 99,868 |
| S47 | nurs* | 1,174,144 |
| S48 | (MH "Physicians+") | 182,335 |
| S49 | physician* | 800,472 |
| S50 | doctor* | 289,224 |
| S51 | S29 OR S30 OR S31 OR S32 OR S33 OR S34 OR S35 OR S36 OR S37 OR S38 OR S39 OR S40 OR S41 OR S42 OR S43 OR S44 OR S45 OR S46 OR S47 OR S48 OR S49 OR S50 | 5,537,776 |
| S52 | (MH "Attitude of Health Personnel+") | 170,687 |
| S53 | TI attitude* OR AB attitude* | 193,675 |
| S54 | TI experience* OR AB experience* | 1,397,630 |
| S55 | TI perception* OR AB perception* | 337,516 |
| S56 | TI perspective* OR AB perspective* | 467,387 |
| S57 | view* | 587,882 |
| S58 | understanding* | 1,206,782 |
| S59 | opinion* | 268,655 |
| S60 | S52 OR S53 OR S54 OR S55 OR S56 OR S57 OR S58 OR S59 | 3,913,449 |
| S61 | S17 AND S28 AND S51 AND S60 | 1,998 |
| S62 | covid-19 or coronavirus or 2019-ncov or sars-cov-2 or cov-19 or covid | 423,169 |
| S63 | s61 NOT s62 | 1,887 |
| S64 | s61 NOT s62 | 1,716 |

| Database: | Academic Search Elite |
| --- | --- |
| Filters: | Language (English, Scandinavian) Peer-reviewed articles |
| Search date: | 08.02.24/27.01.2025 |
| Number of results: | 1022 |

| **#** | **Query** | **Results** |
| --- | --- | --- |
| S1 | DE "ARTIFICIAL respiration" + | 27,764 |
| S2 | artificial n2 respirat* | 16,033 |
| S3 | respirat* | 438,657 |
| S4 | noninvasive ventilat* | 2,438 |
| S5 | ventilat* | 112,938 |
| S6 | vaporizer* | 1,124 |
| S7 | DE "BLOOD pressure testing machines" | 479 |
| S8 | blood pressure device* | 724 |
| S9 | electroencephalograh* | 17 |
| S10 | EEG | 62,644 |
| S11 | medical N2 (equipment OR intervention* OR technolog* OR device*) | 198,515 |
| S12 | electrode* | 313,093 |
| S13 | oxygenator* | 2,131 |
| S14 | DE "OXIMETERS" OR DE "PULSE oximeters" | 1,604 |
| S15 | pulse oximeter* | 4,051 |
| S16 | oximeter* | 4,391 |
| S17 | S1 OR S2 OR S3 OR S4 OR S5 OR S6 OR S7 OR S8 OR S9 OR S10 OR S11 OR S12 OR S13 OR S14 OR S15 OR S16 | 1,094,491 |
| S18 | DE "TERMINAL care" | 11,954 |
| S19 | terminal care | 18,757 |
| S20 | end of life | 99,714 |
| S21 | dying | 41,445 |
| S22 | dying process | 724 |
| S23 | DE "HOSPICE care" | 6,971 |
| S24 | hospice care | 18,665 |
| S25 | DE "PALLIATIVE treatment" | 29,393 |
| S26 | palliative care | 56,403 |
| S27 | terminal* n2 patient* | 5,515 |
| S28 | S18 OR S19 OR S20 OR S21 OR S22 OR S23 OR S24 OR S25 OR S26 OR S27 | 181,145 |
| S29 | DE "CAREGIVERS" | 29,944 |
| S30 | carer* | 43,654 |
| S31 | caregiver* | 80,821 |
| S32 | DE "FAMILIES" | 69,722 |
| S33 | famil* | 1,711,206 |
| S34 | loved one* | 8,128 |
| S35 | partner* | 457,989 |
| S36 | spouse* | 37,787 |
| S37 | significant other | 8,291 |
| S38 | next of kin | 1,144 |
| S39 | relative* | 1,523,870 |
| S40 | DE "HOSPITAL medical staff" | 7,124 |
| S41 | medical staff | 15,887 |
| S42 | healthcare provider* | 54,107 |
| S43 | healthcare professional* | 47,940 |
| S44 | healthcare worker* | 25,027 |
| S45 | DE "NURSES" | 66,624 |
| S46 | nurs* | 758,784 |
| S47 | physician* | 522,068 |
| S48 | doctor* | 356,871 |
| S49 | S29 OR S30 OR S31 OR S32 OR S33 OR S34 OR S35 OR S36 OR S37 OR S38 OR S39 OR S40 OR S41 OR S42 OR S43 OR S44 OR S45 OR S46 OR S47 OR S48 | 4,853,831 |
| S50 | attitude of health personnel | 5,467 |
| S51 | TI attitude* or AB attitude* | 282,996 |
| S52 | TI experience* OR AB experience* | 1,422,305 |
| S53 | TI perception* OR AB perception* | 384,817 |
| S54 | TI perspective* OR AB perspective* | 659,019 |
| S55 | view* | 1,108,836 |
| S56 | understanding* | 1,248,822 |
| S57 | opinion* | 471,997 |
| S58 | S50 OR S51 OR S52 OR S53 OR S54 OR S55 OR S56 OR S57 | 4,728,968 |
| S59 | S17 AND S28 AND S49 AND S58 | 1,157 |
| S60 | covid-19 or coronavirus or 2019-ncov or sars-cov-2 or cov-19 or covid | 339,550 |
| S61 | S59 NOT S60 | 1,088 |
| S62 | S59 NOT S60  Limiters - Peer Reviewed  Expanders - Apply equivalent subjects  Search modes - Boolean/Phrase | 1,040 |
| S63 | S59 NOT S60  Limiters - Peer Reviewed  Expanders - Apply equivalent subjects  Narrow by Language: - english  Search modes - Boolean/Phrase | 1,022 |

| Database: | CINAHL |
| --- | --- |
| Filters: | Language (English, Scandinavian) Peer-reviewed articles |
| Search date: | 09.02.24/27.01.2025 |
| Number of results: | 837 |

| **#** | **Query** | **Results** |
| --- | --- | --- |
| S1 | (MH "Respiration, Artificial+") | 38,728 |
| S2 | artificial n2 respirat* | 26,079 |
| S3 | respirat* | 177,929 |
| S4 | noninvasive ventilat* | 1,275 |
| S5 | ventilat* | 65,857 |
| S6 | vaporizer* | 5,755 |
| S7 | (MH "Blood Pressure Devices+") | 1,821 |
| S8 | blood pressure device* | 1,267 |
| S9 | (MH "Electrical Equipment and Supplies+") | 1,632 |
| S10 | electroencephalograh* OR EEG | 12,843 |
| S11 | medical N2 (equipment OR intervention* OR technolog* OR device*) | 36,589 |
| S12 | (MH "Electrodes+") | 29,076 |
| S13 | electrode* | 17,780 |
| S14 | (MH "Oxygenators+") | 380 |
| S15 | oxygenator* | 803 |
| S16 | (MH "Oximeters+") | 524 |
| S17 | pulse oximeter* | 3,094 |
| S18 | oximeter* | 3,349 |
| S19 | S1 OR S2 OR S3 OR S4 OR S5 OR S6 OR S7 OR S8 OR S9 OR S10 OR S11 OR S12 OR S13 OR S14 OR S15 OR S16 OR S17 OR S18 | 304,041 |
| S20 | (MH "Terminal Care+") | 74,253 |
| S21 | terminal care | 26,654 |
| S22 | end of life | 35,439 |
| S23 | dying | 19,027 |
| S24 | dying process | 753 |
| S25 | (MH "Hospice Care") | 9,893 |
| S26 | hospice care | 24,944 |
| S27 | (MH "Palliative Care") | 42,948 |
| S28 | palliative care | 53,000 |
| S29 | terminal* n2 patient* | 14,919 |
| S30 | S20 OR S21 OR S22 OR S23 OR S24 OR S25 OR S26 OR S27 OR S28 OR S29 | 109,440 |
| S31 | (MH "Caregivers") | 44,402 |
| S32 | carer* | 41,579 |
| S33 | caregiver* | 88,929 |
| S34 | (MH "Family+") | 275,364 |
| S35 | famil* | 469,509 |
| S36 | loved one* | 4,599 |
| S37 | partner* | 114,232 |
| S38 | spouse* | 20,031 |
| S39 | significant other | 4,855 |
| S40 | next of kin | 907 |
| S41 | relative* | 280,621 |
| S42 | (MH "Medical Staff+") | 8,192 |
| S43 | medical staff | 11,790 |
| S44 | healthcare provider* | 75,595 |
| S45 | healthcare professional* | 46,306 |
| S46 | healthcare worker* | 43,085 |
| S47 | (MH "Medical Staff, Hospital+") | 6,771 |
| S48 | (MH "Nurses+") | 231,333 |
| S49 | nurs* | 969,555 |
| S50 | (MH "Physicians+") | 133,440 |
| S51 | physician* | 281,488 |
| S52 | doctor* | 111,869 |
| S53 | S31 OR S32 OR S33 OR S34 OR S35 OR S36 OR S37 OR S38 OR S39 OR S40 OR S41 OR S42 OR S43 OR S44 OR S45 OR S46 OR S47 OR S48 OR S49 OR S50 OR S51 OR S52 | 2,129,054 |
| S54 | (MH "Attitude of Health Personnel+") | 128,787 |
| S55 | TI attitude* OR AB attitude* | 100,448 |
| S56 | TI experience* OR AB experience* | 542,086 |
| S57 | TI perception* OR AB perception* | 156,595 |
| S58 | TI perspective* OR AB perspective* | 176,109 |
| S59 | view* | 155,989 |
| S60 | understanding* | 257,402 |
| S61 | opinion* | 52,245 |
| S62 | S54 OR S55 OR S56 OR S57 OR S58 OR S59 OR S60 OR S61 | 1,199,639 |
| S63 | S19 AND S30 AND S53 AND S62 | 951 |
| S64 | covid-19 or coronavirus or 2019-ncov or sars-cov-2 or cov-19 or covid | 157,564 |
| S65 | S63 NOT S64 | 907 |
| S66 | S63 NOT S64 | 837 |
|  |  |  |

|  |  |  |
| --- | --- | --- |

| Database: | Embase |
| --- | --- |
| Filters: | Language (English, Scandinavian) |
| Search date: | 08.02.24/27.01.2025 |
| Number of results: | 1628 |

1 exp artificial ventilation/ 256269

2 (artificial adj2 respirat*).mp. 2404

3 respirat*.mp. 1371484

4 noninvasive ventilat*.mp. 10037

5 ventilat*.mp. 426800

6 vaporizer*.mp. 2769

7 blood pressure device*.mp. 539

8 (electroencephalograh* or EEG).mp. 147562

9 (medical adj2 (equipment or intervention* or technolog* or device*)).mp. 151699

10 exp electrode/ 205579

11 electrode*.mp. 289003

12 exp oxygenator/ 8280

13 oxygenator*.mp. 8386

14 pulse oximeter*.mp. 9995

15 exp oximeter/ 10807

16 oximeter*.mp. 14149

17 1 or 2 or 3 or 4 or 5 or 6 or 7 or 8 or 9 or 10 or 11 or 12 or 13 or 14 or 15 or 16 2178540

18 exp terminal care/ 87696

19 terminal care.mp. 44593

20 end of life.mp. 49437

21 dying.mp. 59766

22 dying process.mp. 1268

23 exp hospice care/ 13885

24 hospice care.mp. 15777

25 exp palliative therapy/ 146124

26 palliative care.mp. 70304

27 (terminal* adj2 patient*).mp. 16056

28 18 or 19 or 20 or 21 or 22 or 23 or 24 or 25 or 26 or 27 276826

29 exp caregiver/ 118891

30 carer*.mp. 30568

31 caregiver*.mp. 167822

32 exp family/ 617700

33 famil*.mp. 1864108

34 loved one*.mp. 7848

35 partner*.mp. 310153

36 spouse*.mp. 34520

37 significant other.mp. 2246

38 next of kin.mp. 2769

39 relative*.mp. 2111490

40 exp medical staff/ 42991

41 medical staff.mp. 54928

42 healthcare provider*.mp. 52020

43 healthcare professional*.mp. 60546

44 healthcare worker*.mp. 29810

45 exp nurse/ 222918

46 nurs*.mp. 903759

47 exp physician/ 996441

48 physician*.mp. 864844

49 doctor*.mp. 334294

50 29 or 30 or 31 or 32 or 33 or 34 or 35 or 36 or 37 or 38 or 39 or 40 or 41 or 42 or 43 or 44 or 45 or 46 or 47 or 48 or 49 6525341

51 exp health personnel attitude/ 208158

52 attitude*.m_titl. 77856

53 attitude*.ab. 223968

54 experience*.m_titl. 420000

55 experience*.ab. 1727173

56 perception*.m_titl. 115021

57 perception*.ab. 375034

58 perspective*.m_titl. 200255

59 perspective*.ab. 404119

60 view*.mp. 719662

61 understanding*.mp. 1432717

62 opinion*.mp. 191132

63 51 or 52 or 53 or 54 or 55 or 56 or 57 or 58 or 59 or 60 or 61 or 62 4875615

64 17 and 28 and 50 and 63 3123

65 (covid-19 or coronavirus or 2019-ncov or sars-cov-2 or cov-19 or covid).mp. 514671

66 64 not 65 2870

67 limit 66 to ((danish or english or norwegian or swedish) and (article or "review")) 1628

| Database: | Cochrane |
| --- | --- |
| Filters: | Cochrane Reviews |
| Search date: | 12.02.24/27.01.2025 |
| Number of results: | 74 |

ID Search Hits

#1 ("terminal care"):ti,ab,kw in Cochrane Reviews 11

#2 (hospice):ti,ab,kw in Cochrane Reviews 9

#3 ("end of life"):ti,ab,kw in Cochrane Reviews 28

#4 (dying):ti,ab,kw in Cochrane Reviews 215

#5 ("palliative care"):ti,ab,kw in Cochrane Reviews 81

#6 #1 OR #2 OR #3 OR #4 OR #5 298

#7 (attitude*):ti,ab,kw in Cochrane Reviews 117

#8 (experience*):ti,ab,kw in Cochrane Reviews 1874

#9 (perspective*):ti,ab,kw in Cochrane Reviews 82

#10 #7 OR #8 OR #9 2006

#11 #6 AND #10 74

A free text and phrase search was conducted on a limited number of key terms, focusing on the experiential component of the study and the terminal phase. The inclusion of all search terms, as per the searches in the other databases, resulted in around 2500 irrelevant hits. It was therefore decided that only a small number of terms would be included, making irrelevant reviews easier to eliminate during screening.
